# Effects of Water Column Mixing and Stratification on Planktonic Primary Production and Dinitrogen Fixation on a Northern Red Sea Coral Reef

**DOI:** 10.3389/fmicb.2018.02351

**Published:** 2018-10-01

**Authors:** Arjen Tilstra, Nanne van Hoytema, Ulisse Cardini, Vanessa N. Bednarz, Laura Rix, Malik S. Naumann, Fuad A. Al-Horani, Christian Wild

**Affiliations:** ^1^Marine Ecology Department, Faculty of Biology and Chemistry, University of Bremen, Bremen, Germany; ^2^Coral Reef Ecology Group, Leibniz Centre for Tropical Marine Research, Bremen, Germany; ^3^Department of Integrative Marine Ecology, Stazione Zoologica Anton Dohrn, Naples, Italy; ^4^Centre Scientifique de Monaco, Monaco, Monaco; ^5^School of Biological Sciences, The University of Queensland, Brisbane, QLD, Australia; ^6^Marine Science Station, The University of Jordan, Amman, Jordan

**Keywords:** plankton, primary production, dinitrogen fixation, Gulf of Aqaba, diazotrophs

## Abstract

The northern Red Sea experiences strong annual differences in environmental conditions due to its relative high-latitude location for coral reefs. This allows the study of regulatory effects by key environmental parameters (i.e., temperature, inorganic nutrient, and organic matter concentrations) on reef primary production and dinitrogen (N_2_) fixation, but related knowledge is scarce. Therefore, this study measured environmental parameters, primary production and N_2_ fixation of phytoplankton groups in the water overlying a coral reef in the Gulf of Aqaba. To this end, we used a comparative approach between mixed and stratified water column scenarios in a full year of seasonal observations. Findings revealed that inorganic nutrient concentrations were significantly higher in the mixed compared to the stratified period. While gross photosynthesis and N_2_ fixation rates remained similar, net photosynthesis decreased from mixed to stratified period. Net heterotrophic activity of the planktonic community increased significantly during the stratified compared to the mixed period. While inorganic nitrogen (N) availability was correlated with net photosynthesis over the year, N_2_ fixation only correlated with N availability during the mixed period. This emphasizes the complexity of planktonic trophodynamics in northern Red Sea coral reefs. Comparing mixed and stratified planktonic N_2_ fixation rates with those of benthic organisms and substrates revealed a close seasonal activity similarity between free-living pelagic and benthic diazotrophs. During the mixed period, N_2_ fixation potentially contributed up to 3% of planktonic primary production N demand. This contribution increased by ca. one order of magnitude to 21% during the stratified period. Planktonic N_2_ fixation is likely a significant N source for phytoplankton to maintain high photosynthesis under oligotrophic conditions in coral reefs, especially during stratified conditions.

## Introduction

Coral reefs thrive under oligotrophic conditions, particularly due to autochthonous generation of organic carbon (C) and nitrogen (N) through photosynthetic primary production, prokaryotic dinitrogen (N_2_) fixation and efficient internal recycling of those materials within the ecosystem (Hatcher, 1997). Internal recycling can occur through strong benthic-pelagic coupling of dissolved and particulate organic matter (POM) and nutrients (Yahel et al., 1998; Wild et al., 2004; Chipman et al., 2012). Benthic-pelagic coupling is mediated by benthic organisms such as corals, algae, and sponges (Haas et al., 2010; Naumann et al., 2010; de Goeij et al., 2013), but also by reef sediment and framework microbiota (Rasheed et al., 2002; Wild et al., 2009). Furthermore, reefs receive allochthonous energy and nutrients in the form of inorganic nutrients, plankton, and particulate/dissolved matters from offshore and/or riverine inflow. Since reefs are often N limited, diazotrophs (i.e., prokaryotes capable of N_2_ fixation) can facilitate primary production, particularly under oligotrophic conditions such as those found in the Gulf of Aqaba (Capone et al., 1997; Karl et al., 2002; Furnas et al., 2011).

The Gulf of Aqaba forms one of the northern tips of the Red Sea. Its desert coasts are bordered by fringing coral reefs that experience relatively strong variation in light availability and water temperature for warm-water coral reefs due to its relative high latitude location. The annual fluctuation in sea surface temperature (21–29°C) combined with relatively warm deeper water layers (year round ∼21°C for water depth >200 m) result in an annual cycle of deep water mixing from December until May and stratification down to 200 m water depth from June until ∼November (Carlson et al., 2014) with slow destratification from October and onwards (Biton and Gildor, 2011a,b,c). Inorganic nutrients are brought up to surface water layers during deep water mixing but are trapped in deeper waters during stratification, creating extreme oligotrophic conditions in coral reef surrounding surface waters (Rasheed et al., 2002, 2012; Silverman et al., 2007). These local physico-chemical conditions offer the rare opportunity to study the effects of variation in key environmental factors on important processes such as primary production and diazotrophy within coral reefs.

Planktonic primary production in the Gulf of Aqaba and northern Red Sea is dominated by photoautotrophic nano- and picoplankton. The plankton fraction <20 μm performs on average 81% of planktonic photosynthesis in the upper water layer (discrete depth) (Qurban et al., 2014) ranging from 0.02 to 3.38 μmol C L^-1^ d^-1^ (Levanon-Spanier et al., 1979; Qurban et al., 2014), assuming a 12 h period of daylight. The phytoplankton community in the Gulf of Aqaba is characterized by a strong shift in its composition between annual periods, i.e., mixed and stratified. During the mixed period, eukaryotic algae account for up to 95% of phytoplankton biomass, while during stratification >60% of the biomass consists of prokaryotes, in particular *Prochlorococcus* sp. which may comprise up to 50% of the biomass (Al-Najjar et al., 2007). The main groups of planktonic diazotrophs in the Gulf of Aqaba are Cyanobacteria and Proteobacteria, and they are responsible for water column N_2_ fixation rates ranging from 0.01 to 1.9 nmol N L^-1^ d^-1^ (Foster et al., 2009; Rahav et al., 2015). Planktonic photosynthesis rates in reef-surrounding waters can be 1–2 orders of magnitude higher than in oceanic waters offshore (D’Elia and Wiebe, 1990; Adey, 1998). Moreover, primary production and N_2_ fixation can interact synergistically within or between planktonic organisms; photosynthesis (and the subsequent organic C released to the surrounding water) may fuel the energy-demanding process of N_2_ fixation, which in turn can support primary production by supplying the bioavailable N required to synthesize proteins for photosynthesis (e.g., Foster et al., 2011).

Seasonality in the Gulf of Aqaba affects benthic primary production and N_2_ fixation in a range of common organisms and substrates including corals (Bednarz et al., 2015a; Cardini et al., 2015), algae (Rix et al., 2015; Tilstra et al., 2017), and sediments (Bednarz et al., 2015b). Some of these studies found positive relationships between the two processes, as well as with light intensity, temperature and a negative relationship with N availability (Bednarz et al., 2015a; Rix et al., 2015; Tilstra et al., 2017). While studies exist for open water planktonic primary production and/or N_2_ fixation in the Gulf of Aqaba (e.g., Foster et al., 2009; Rahav et al., 2015; Shiozaki et al., 2018), to our knowledge, there are no studies relating these two processes of planktonic communities directly overlying a coral reef, where strong benthic pelagic coupling is evident. Thus, to increase our understanding of the factors regulating planktonic primary production and N_2_ fixation, the objectives of this study were (i) to measure environmental parameters in a Gulf of Aqaba fringing coral reef over the two distinct periods (i.e., mixed and stratified), (ii) to quantify primary production and N_2_ fixation of the planktonic community, in water directly overlying a coral reef, over the two periods, (iii) to investigate functional relationships between primary production, N_2_ fixation and environmental parameters, and (iv) to compare the pattern of primary production and N_2_ fixation with benthic organisms and substrates investigated in parallel.

## Materials and Methods

### Study Site and Environmental Monitoring

The fieldwork for this study was conducted at the Marine Science Station (MSS) of The University of Jordan, located 10 km south of Aqaba, Jordan. The MSS is situated adjacent to a marine protected area encompassing a crescent shaped fringing coral reef with a length of ca. 1 km. All water column sampling was performed at 10 m water depth in the fore reef section (29° 27′ 31″ N, 34° 58′ 26″ E). Two extensive fieldwork campaigns were performed in 2013: one during the mixed period (January–April; 12 weeks), and one during the stratified period (September–November; 13 weeks). The studied reef consists of a shallow reef flat (<1 m water depth) surrounded by a carbonate sediment belt at ca. 5 m water depth and a coral dominated middle-fore reef facing the open sea [see Cardini et al. (2016) for a visual description of the site].

Light intensity, water temperature, chlorophyll *a* (Chl *a*), dissolved organic carbon (DOC), particulate organic carbon (POC), particulate nitrogen (PN), and inorganic nutrients, i.e., NH_4_^+^, PO_4_^3-^, NO_2_^-^ and NO_3_^-^, were monitored during each period (at 10 m water depth). Light intensity measurements recorded by data loggers in lux units were converted to photosynthetically active radiation (PAR) by a conversion factor calculated from a simultaneous minute-by-minute measurement of lux and PAR (08:00–14:00 on 1 day, *n* = 353) using a HOBO pendant logger and a LI-COR LI192SA underwater quantum sensor: lux = PAR x 52.0, *R*^2^ = 0.83. This value is comparable to the conversion factor given by Valiela (1984): 51.2. PAR values measured per minute were summed for each weekly water-sampling day: values in μmol quanta m^-2^ s^-1^ were recalculated to mol quanta m^-2^ d^-1^. Water temperature, measured per minute on the water-sampling day, was averaged over the 24 h period. Water samples were collected from 10 m water depth on a weekly basis (during both campaigns) in clean high density poly-ethylene (HDPE) containers (volume: 5 L) using SCUBA. All samples were collected between 08:00 and 10:00 within a 10 min timeframe. Sampling was performed 1 m above the seafloor without disturbing the benthos. Chl *a* and NH_4_^+^ were measured fluorometrically while remaining parameters (i.e., POC, PN, PO_4_^3-^, NO_2_^-^, and NO_3_^-^) were measured photometrically as detailed by Bednarz et al. (2015b). In addition, DOC was measured according to Cardini et al. (2015) using a Shimadzu TOC-VCPH total organic carbon analyser. For temperature, PAR, nutrient (DIN and phosphate) and chl *a* data along the entire depth gradient, see Bednarz et al. (2018).

### Quantification of Primary Production

All incubations for primary production and N_2_ fixation rates were performed in a flow through mesocosm (∼4000 l h^-1^ exchange rate) with water from the reef at 10 m sampling depth to ensure *in situ* conditions (temperature). Light intensity was adjusted to 10 m depth values by using black netting.

Two water subsamples were taken per HDPE container, one for net photosynthesis (*P*_n_) and one for dark respiration (*R*) measurement. Weekly incubations were performed in individual 1 L closed cell respirometric glass chambers (*n* = 6) under constant stirring (600 rpm). Chambers for *R* measurements were placed in bags made of dense opaque plastic (volume: 10 L) for incubation in the dark. *P*_n_ incubations were performed from 10:00 until sunset (ca. 17:00–18:00; depending on the period), while *R* incubations ran for 24 h to get measurable rates and to account for diurnal fluctuations. *P_n_* and *R*-values were obtained by using a conductivity- and temperature-corrected O_2_ optode sensor (MultiLine^®^ IDS 3430, WTW, accuracy: ± 0.5% of measured value) and calculated by subtracting O_2_ start from end concentrations. Subsequently, values obtained for *P*_n_ and *R* were corrected for incubation duration and chamber volume, and recalculated to molar equivalents resulting in hourly O_2_ measurements in μmol O_2_ L^-1^ h^-1^. *R* is presented here as a positive value. Estimates of gross photosynthesis (*P*_g_) were calculated as *P*_g_ = *P*_n_ + *R*. As *R* is averaged over 24 h, it represents a value assuming a constant hourly rate. Since *R* is susceptible to daily fluctuations, with possible lower *R* during darkness, *P*_g_-values are possibly underestimated. However, *R*-values measured here also includes the heterotrophic component of the community adding to *P*_g_.

To assess the contribution of planktonic C to the water column and C budget of the ecosystem, *P*_n_ and *R* rates were recalculated to metabolic C production per day by the following equations, assuming photosynthetic and respiratory quotients of 1.4 and 1.1, respectively (McKinnon et al., 2013). Gross primary production (GPP) = (*P*_n_ + *R*) × h of daylight (using the average hourly rate of *R*), calculates the daily fixation of C by autotrophs; community respiration (CR) = *R* × 24 h, calculates daily respiration of the entire community, i.e., auto- and heterotrophs; net community production (NCP) = GPP–CR, calculates whether the system is net autotrophic or heterotrophic over the 24 h. Values presented as μmol C L^-1^ d^-1^. Finally, daily contribution of planktonic C to total organic C (TOC = DOC + POC) was calculated by dividing GPP, NCP, or CR with TOC, and multiplying by 100 to obtain percentages.

### Quantification of N_2_ Fixation

Planktonic N_2_ fixation rates were measured during the mixed and stratified periods using a modified acetylene (C_2_H_2_) reduction technique (Capone, 1993; Wilson et al., 2012). Weekly incubations were performed in 1 L chambers (*n* = 8) containing 800 mL seawater and a 200 mL headspace both being 10% C_2_H_2_-enriched under constant stirring (600 rpm). Control incubations were performed without C_2_H_2_ addition to measure biological ethylene (C_2_H_4_) production, as well as with sterile filtered seawater (0.2 μm) to measure inorganic C_2_H_4_ production from C_2_H_2_. The incubations lasted for 24 h, and 1 mL gas samples were extracted from a port in the lid at 0, 4, and 24 h with a gastight syringe and analyzed for C_2_H_4_ concentration using a customized reducing compound photometer (Peak Laboratories, Mountain View, CA, United States, detection limit = 100 ppb). C_2_H_4_ measurements were recalculated to nmol C_2_H_4_ in the whole chamber water volume. It was determined in pilot experiments that the C_2_H_2_ concentration equilibrated between headspace and incubation water in the first 4 h, the C_2_H_4_ production over 4 to 24 h were therefore used for calculations. Changes in C_2_H_4_ concentration over time were corrected for incubation duration and volume of water in the chamber, resulting in measurements of nmol C_2_H_4_ L^-1^ h^-1^. Due to the ongoing debate concerning conversion factors, C_2_H_4_ evolution rates measured here are used as a proxy for estimated N_2_ fixation rates and are thus not converted to actual nitrogen fixed (Wilson et al., 2012). Only for estimating the contribution of N_2_ fixation to the N requirement of primary production, C_2_H_4_ rates were converted to N m^-3^ d^-1^ using the conservative theoretical C_2_H_4_:N_2_ conversion ratio of 4:1 (Mulholland et al., 2004).

### Statistical Analyses

The values of replicate measurements for environmental parameters, primary production and N_2_ fixation for each week were averaged prior to statistical analyses. All statistics were performed in Sigmaplot 12.0 for Windows (Systat software). Environmental parameters, primary production and N_2_ fixation during the two periods were tested for normality with the Shapiro–Wilk test. Comparisons between the two periods were performed with independent samples *T*-tests if data were normally distributed and with Mann–Whitney *U*-tests if data lacked normality.

In addition, relationships between N_2_ fixation rates, *P*_n_, *P*_g_, and *R* rates per period and across both periods with environmental water parameters were determined via linear regression. Differences were deemed significant at *p* < 0.05. All values are given as mean ± SE.

## Results

### Environmental Variables

**FIGURE 1:**
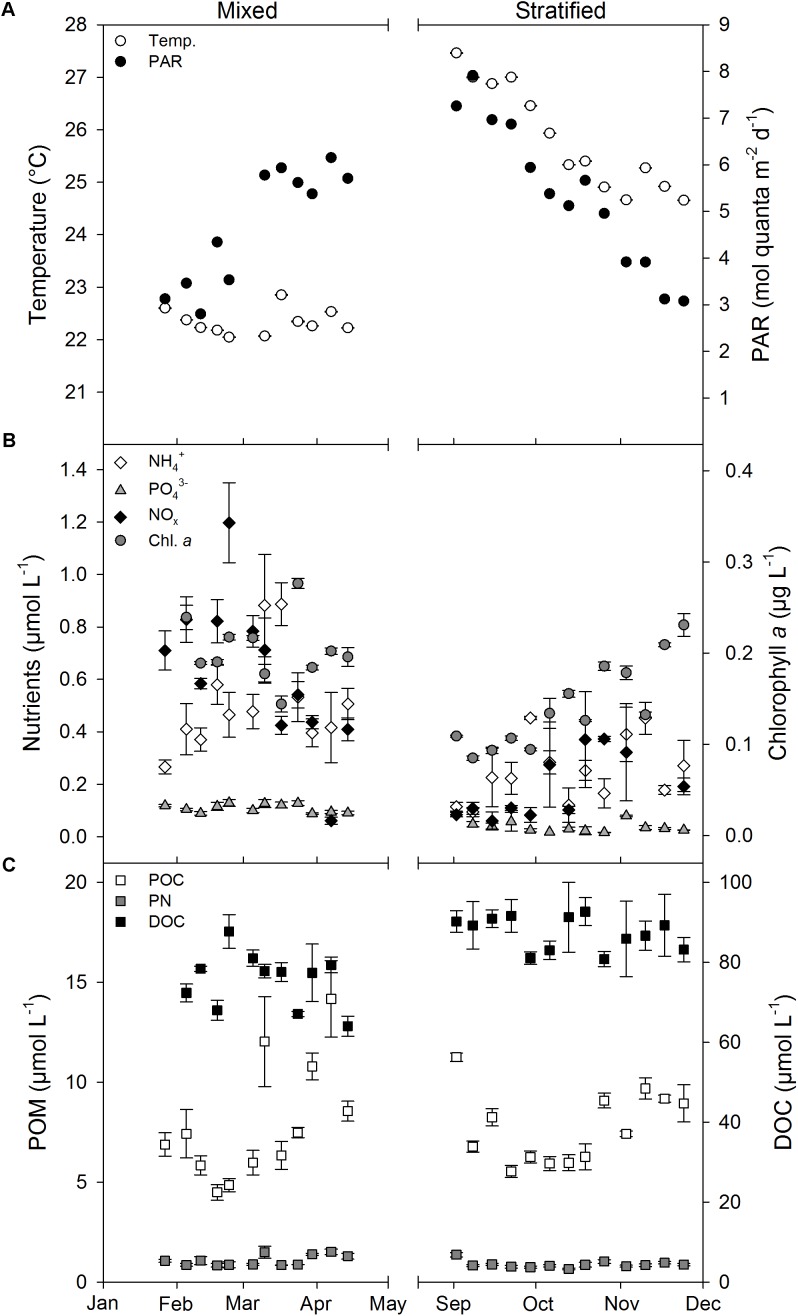
Mean weekly environmental water parameters measured at 10 m water depth (1 m over coral reef benthos) during the mixed and stratified periods. **(A)** Temperature and PAR. **(B)** Nutrients and chlorophyll *a*. **(C)** POM and DOC. Values given as mean ± SE. Temp, water temperature; PAR, photosynthetically active radiation measured at 10 m water depth; NO_x_, NO_2_^-^ + NO_3_^-^; POC, particulate organic carbon; PN, particulate nitrogen; POM, particulate organic matter (POC and PN); DOC, dissolved organic carbon.

Mean weekly measurements of environmental parameters were variable over time (**Figure 1**). Daily PAR increased from January to April (i.e., mixed period) from 3.45 ± 0.26 to 5.76 ± 0.11 mol quanta m^-2^ d^-1^, and decreased again from September to November (i.e., stratified period) from 6.72 ± 0.37 to 4.25 ± 0.38 mol quanta m^-2^ d^-1^ (**Figure 1A**). However, between periods no significant differences were found for PAR (**Table 1**). Temperature was stable throughout the mixed period (22.1–22.9°C), but increased to a maximum of 27.5°C in early September followed by a decrease to 24.7°C at the end of November. Inorganic nutrient and Chl *a* concentrations were all significantly lower during the stratified than during the mixed period (**Figure 1B** and **Table 1**, all *p* < 0.002), while DOC was significantly higher during the stratified period than during the mixed period (**Figure 1C** and **Table 1**). POC concentrations showed no significant differences between periods, while PN was significantly lower in the stratified period (**Figure 1C**), causing a significantly higher POC:PN ratio (**Table 1**). The DIN:PO_4_^3-^ ratio was not significantly different between periods (**Table 1**).

**Table 1:** Environmental water parameters in the mixed and stratified periods.

Parameters	Mixed	Stratified	*p*
PAR (mol m^-2^ d^-1^)	4.71 ± 0.38	5.39 ± 0.44	0.264
Temperature (°C)^∗^	22.34 ± 0.07	25.84 ± 0.28	**<0.001**
Chl *a* (μg L^-1^)	0.20 ± 0.01	0.14 ± 0.01	**0.002**
DOC (μmol L^-1^)	75.51 ± 2.10	87.36 ± 1.17	**<0.001**
POC (μmol L^-1^)	7.90 ± 0.86	7.74 ± 0.50	0.865
TOC (μmol L^-1^)^∗^	83.51 ± 2.32	95.10 ± 1.22	**<0.001**
PN (μmol L^-1^)^∗^	1.08 ± 0.08	0.88 ± 0.05	**0.024**
NH_4_^+^ (μmol L^-1^)^∗^	0.52 ± 0.06	0.25 ± 0.03	**<0.001**
PO_4_^3-^ (μmol L^-1^)	0.11 ± 0.00	0.04 ± 0.01	**<0.001**
NO_x_ (μmol L^-1^)	0.63 ± 0.08	0.19 ± 0.04	**<0.001**
POC:PN	7.16 ± 0.39	8.77 ± 0.34	**0.005**
DIN:PO_4_^3-∗^	10.42 ± 0.70	16.59 ± 3.14	0.717

*Differences were tested with independent sample t-tests, except for parameters with ^∗^, these were tested with Mann–Whitney U-tests due to lack of normality. Chl a, chlorophyll a; DOC, dissolved organic carbon; POC, particulate organic carbon; TOC, total organic carbon (POC + DOC); PN, particulate nitrogen; NOx, NO_2_^-^ + NO_3_^-^; DIN, NH_4_^+^ + NO_x_. Values are given as mean ± SE. Bold p-values indicate significant values.*

### Primary Production

**FIGURE 2:**
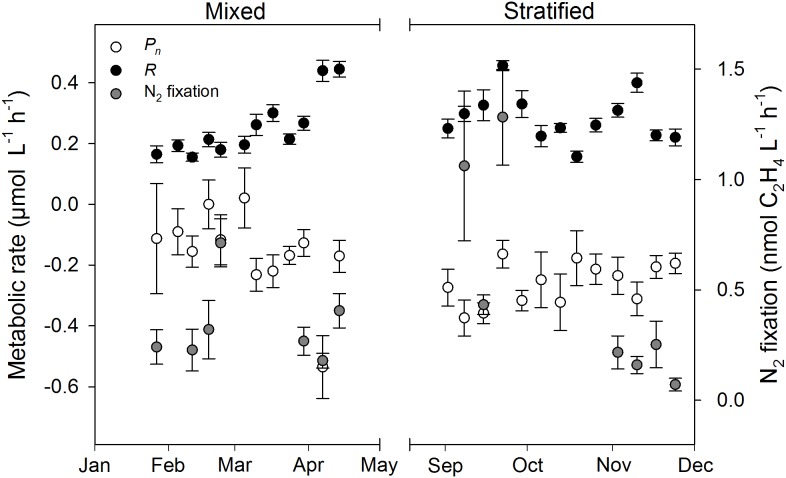
Planktonic primary production and N_2_ fixation rates measured weekly at 10 m water depth (1 m above the benthos) during the mixed and stratified periods. Net photosynthesis (*P*_n_), dark respiration (*R*). Values given as mean ± SE.

**FIGURE 3:**
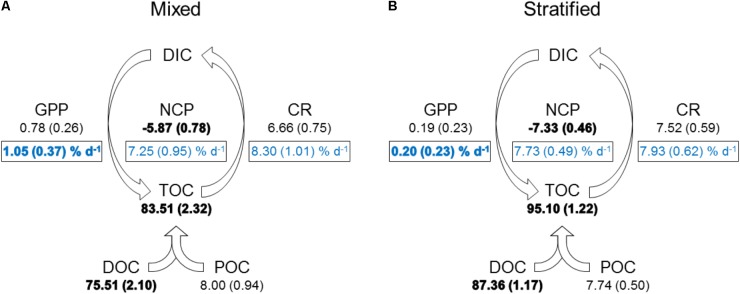
Mean daily planktonic carbon balance in mixed **(A)** and stratified **(B)** periods. GPP, gross primary production; CR, community respiration; NCP, net community production (GPP–CR). Values given as mean (SE). GPP, CR, NCP in μmol C L^-1^ d^-1^. DOC, dissolved organic carbon; TOC, total organic carbon [POC (particulate organic carbon) + DOC], TOC, DOC, and POC in μmol C L^-1^; DIC, dissolved inorganic carbon. Boxed light blue text constitutes the percentage of planktonic C contributed daily to TOC [mean (SE)]. Bold indicates a significant difference between periods (*p* < 0.05).

Mean water column *P*_n_ was always negative (-0.54–-0.09 μmol O_2_ L^-1^ h^-1^), except on 18 February and 3 March (0.00 and 0.02 μmol O_2_ L^-1^ h^-1^, respectively), and significantly lower during the stratified period than the mixed period (**Figure 2** and **Table 2**). *R* ranged from 0.15 to 0.46 μmol O_2_ L^-1^ h^-1^ and was not significantly different between periods. Estimates of *P*_g_ were higher for the mixed period compared to the stratified period with averages of 0.09 and 0.02 μmol O_2_ L^-1^ h^-1^, respectively. However, these estimates were not significantly different (**Table 2**). GPP and CR was relatively stable and not significantly different between periods (**Table 2** and **Figure 3**). Mean water column TOC was 83.51 and 95.10 μmol L^-1^ d^-1^ for the mixed and stratified periods, respectively (**Table 1**). The contribution of net daily planktonic C (NCP) to TOC was comparable during the mixed and stratified periods, 7.25 ± 0.95, and 7.73 ± 0.49% d^-1^, respectively (**Figure 3**).

**Table 2:** Primary production (*P_n_*, *R* and *P_g_*, GPP, CR and NCP) and N_2_ fixation in the mixed and stratified periods.

Parameters	Mixed	Stratified	*p*
*P_n_* (μmol O_2_ L^-1^ h^-1^)^∗^	-0.16 ± 0.04	-0.26 ± 0.02	**0.004**
*R* (μmol O_2_ L^-1^ h^-1^)^∗^	0.25 ± 0.03	0.29 ± 0.02	0.165
*P_g_* (μmol O_2_ L^-1^ h^-1^)	0.09 ± 0.03	0.02 ± 0.03	0.094
GPP (μmol C L^-1^ d^-1^)	0.78 ± 0.26	0.19 ± 0.23	0.094
CR (μmol C L^-1^ d^-1^)^∗^	6.66 ± 0.75	7.52 ± 0.59	0.165
NCP (μmol C L^-1^ d^-1^)^∗^	-5.87 ± 0.78	-7.33 ± 0.46	**0.036**
N_2_ fixation (nmol C_2_H_4_ L^-1^ h^-1^)	0.34 ± 0.07	0.50 ± 0.18	0.423

*Differences were tested with independent sample t-tests, except for parameters with ^∗^, these were tested with Mann–Whitney U-tests due to lack of normality. P_*n*_, net photosynthesis; R, dark respiration; P_g_, gross photosynthesis = P_n_ + R; GPP, gross primary production; CR, community respiration; NCP, net community production over the day = GPP–CR. Values are given as mean ± SE. Bold p-values indicate p< 0.05*.

### N_2_ Fixation Activity

Weekly mean N_2_ fixation ranged from 0.18 to 0.71 and 0.07 to 1.28 nmol C_2_H_4_ L^-1^ h^-1^ in the mixed and stratified periods, respectively (**Figure 2**). Although mean N_2_ fixation on 8 and 22 September, during stratification, were substantially higher than during the mixed period, overall there was no significant difference (**Table 2**).

### Relationship Analyses

Positive relationships were revealed for *P*_n_ and availability of N (NO_x_ and/or DIN) during the mixed and stratified period and when both periods were combined (**Table 3**). Additionally, negative relationships were revealed for *P*_n_ with temperature and PAR. *R* revealed a negative relationship with NO_x_ during the mixed period and when both periods are combined (**Table 3**). No relationships were found for *P*_g_ with any environmental parameter (**Table 3**). Positive relationships for N_2_ fixation and N availability were found for the mixed period while for the stratified period positive relationships were established with both temperature and PAR. A positive relationship was established for PAR when periods were combined (**Table 3**). Additionally, no relationships were found between any of the primary production parameters and N_2_ fixation (not shown in **Table 3**).

**Table 3:** Linear regression analysis (*r*^2^-values) examining the relationships between the physiological parameters.

	Temp	PAR	NOx	DIN
*P*_n_				
Mixed	0.178	0.307	**0.510**^∗^	**0.377**^∗^
Stratified	0.177	0.186	**0.377**^∗^	0.324
Combined	*0.208*^∗^	*0.255*^∗^	**0.544**^∗∗∗^	**0.395**^∗∗^
*R*				
Mixed	0.038		*0.550*^∗∗^	0.257
Stratified	0.133		0.252	0.117
Combined	0.060		*0.327*^∗∗^	0.168
*P*_g_				
Mixed	0.063	0.001	0.073	0.064
Stratified	0.000	0.013	0.003	0.022
Combined	0.082	0.014	0.147	0.148
N_2_ fixation				
Mixed	0.507	0.038	**0.573**^∗^	**0.640**^∗^
Stratified	**0.716**^∗^	**0.700**^∗^	0.302	0.409
Combined	0.248	**0.406**^∗^	0.016	0.057

*P_n_, net photosynthesis; R, dark respiration; P_g_, gross photosynthesis; N_2_ fixation, dinitrogen fixation; Temp, temperature; PAR, photosynthetically active radiation; DIN, dissolved inorganic nitrogen; NO_x_, NO_2_^-^ + NO_3_^-^; DIN, NH_4_^+^ + NO_x_. Bold values indicate significant positive relationships, and italicized values indicate significant negative relationships. ^∗^p < 0.05, ^∗∗^p < 0.01, ^∗∗∗^p < 0.001.*

## DISCUSSION

Studies relating planktonic N_2_ fixation and primary production in the highly seasonal Gulf of Aqaba in the northern Red Sea are, to our knowledge, only related to open water settings (Rahav et al., 2015). Rahav et al. (2015) highlighted the need for both spatial and temporal sampling of planktonic communities in the Gulf of Aqaba to disentangle the complex dynamics of these communities. In this study, water samples taken 1 m above the reef benthos were used to measure planktonic primary production and N_2_ fixation rates on a weekly basis during both the mixed and the stratified period. Findings revealed significantly higher primary production (*P*_n_) during the mixed period (**Table 2**), while planktonic N_2_ fixation rates remained stable between both periods (**Table 2**). Similar to open water, higher net heterotrophic activity was measured during the stratified compared to the mixed period, but the open water column remained net autotrophic. Moreover, Rahav et al. (2015) found a potential coupling between pelagic primary production and N_2_ fixation only during the mixed period, while linear regression analyses supported no such relationship here. However, results obtained here do suggest that N_2_ fixation, as estimated by C_2_H_4_ evolution, has the potential to maintain stable pelagic GPP throughout the year by contributing ∼6 times more bioavailable N for GPP during the N depleted stratified period compared to the mixed period.

### Environmental Parameters in Mixed and Stratified Period

Photosynthetically active radiation, water temperature, and inorganic nutrient concentrations during the mixed and stratified periods were comparable to previous research at the study site (Rasheed et al., 2002; Naumann et al., 2010). The average mixed period DIN:PO_4_^3-^ ratio (10.42) was lower than the Redfield ratio for N:P [16:1; Redfield (1958)], suggesting N as the limiting nutrient. The average N:P ratio in the stratified period was similar to the Redfield N:P ratio, indicating that inorganic N and P availability in that period was on average balanced. However, ratios between 31 and 33 found in three non-consecutive weeks in October and November suggest that there were times when P may have been limiting. Previous studies have also found that both N and P are limiting factors for primary production in the northern Red Sea (Stihl et al., 2001; Mackey et al., 2007; Suggett et al., 2009). Chl *a* decreased significantly from the mixed to the stratified period, coinciding with declining inorganic nutrients and increasing water temperature and PAR. A strong negative relationship with PAR was found especially during the stratified period (*r*^2^ = 0.776, *p* < 0.001). Indeed, higher PAR often causes a reduction in Chl *a* in phytoplankton (Laabir et al., 2011), but this response is specific per species (Kulk et al., 2011). Furthermore, Gittings et al. (2018) found a strong, near perfect, negative correlation between Chl *a* and sea surface temperature in the northern Red Sea. This is also confirmed by our data as a strong negative relationship was found between Chl *a* and temperature when both periods were combined (*r*^2^ = 0.597, *p* < 0.001). Average POC:PN ratios in the mixed and stratified period were higher than Redfield proportions (106:16 = 6.625), 7.16 and 8.77, respectively, suggesting that the POM in the water column was impoverished in N throughout the year, particularly so during the stratified period when PN concentrations were significantly reduced. The dominant source of POM in coral reef-surrounding waters can be mucus released by hard corals (Johannes, 1967; Naumann et al., 2012a). Mucus POC and PN release by the dominant hard corals in the studied reef is constant over the year (Naumann et al., 2010). However, the average POC:PN ratio of coral mucus (12 ± 1; Naumann et al., 2010), is far higher than the periodic ratios found for our water samples, indicating that a large fraction of water column PN originated from another source subject to differences in environmental conditions. This is confirmed by Hadas et al. (2009), who found that the majority of water column PN in a Gulf of Aqaba reef consisted of or was produced by pelagic prokaryotes. In addition, DOC measured in our water samples was significantly higher in the stratified period (87.36 μmol L**^-^**^1^) compared to the mixed period (75.51 μmol L**^-^**^1^). DOC may be more abundantly released by benthic coral reef algae than by co-occurring hard corals (Haas et al., 2013; Mueller et al., 2014). DOC release by turf algae and the algal genus *Peyssonnelia* in the studied reef is indeed higher during the stratified than during the mixed period (Haas et al., 2010). A positive relation between DOC release and temperature is common in marine macrophytes (e.g., Maher and Eyre, 2010). Thus, increased benthic release could explain the increased water column DOC concentration found during the stratified period.

### Planktonic Primary Production and N_2_ Fixation

The planktonic GPP rates measured in the mixed and stratified periods fall within the range previously recorded for the Gulf of Aqaba (Levanon-Spanier et al., 1979) and rates measured for other reef-surrounding waters worldwide (**Table 4**). However, compared to open surface waters of the Gulf of Aqaba, GPP found here was three- to fourfold higher during the mixed period and four- to ninefold higher during the stratified period (Rahav et al., 2015). NCP was always negative, indicating that the planktonic community as a whole acted net heterotrophically, particularly during the stratified period (Cardini et al., 2016). Planktonic communities in waters surrounding coral reefs are often net heterotrophic and are likely fueled by a steady supply of organic matter released from the reef benthos (Haas et al., 2013; Naumann et al., 2012b). Even though PAR and temperature were both negatively related with *P*_n_, the strong positive relationship of *P*_n_ with both DIN and/or NO_x_ within each period and combined indicated that inorganic N, rather than PAR or temperature, was the strongest environmental parameter that limited *P*_n_ in these oligotrophic waters.

**Table 4:** Comparison of daily gross primary production (GPP) rates from this study and literature references.

Location	GPP (μmol C L^-1^ d^-1^)	Reference
Gulf of Aqaba	0.00–2.54^†^	This study
Gulf of Aqaba	0.05–3.38^∗^	Levanon-Spanier et al., 1979
Gulf of Aqaba (open surface water)	0.02–0.26	Rahav et al., 2015
Northern Red Sea	0.02–3.00^∗^	Qurban et al., 2014
Great Barrier Reef, Australia	0.80–3.33	Sorokin, 1995
Vietnam	0.15–3.00	Tac-An et al., 2013
Hawaii	2.01 ± 0.17^†^ (mean ± SE)	Johnson and Wiegner, 2014
New Caledonia	0.47 ± 0.05 (mean ± SE)	Torréton et al., 2010

*^†^Recalculated from O_2_ values using a photosynthetic quotient of 1.4 (McKinnon et al., 2013). ^∗^extrapolated to per day assuming 12 h of daylight*.

Dinitrogen fixation rates measured in this study were within the range found for planktonic communities from different locations worldwide (**Table 5**). Our maximum rates appear high compared to most literature values, but this is mainly due to two high values measured in September (1.06 and 1.28 nmol C_2_H_4_ L^-1^ h^-1^). The other weekly mean values in the present study average 0.29 ± 0.05 nmol C_2_H_4_ L^-1^ h^-1^, which falls within the range of most literature values (0.004–0.46 nmol C_2_H_4_ L^-1^ h^-1^). These values, like the values in this study, were found in the upper water column up to 10 m depth, while higher values (∼2.75 and 0.87 nmol C_2_H_4_ L^-1^ h^-1^) were measured at depths >25 m (Wilson et al., 2012; Wu et al., 2018). Measured values from the tropical Atlantic Ocean [up to 1.04 C_2_H_4_ L^-1^ h^-1^ (Großkopf et al., 2012), assuming the conservative theoretical C_2_H_4_:N_2_ conversion ratio of 4:1 (Mulholland et al., 2004)], indicate that high rates, such as those found in September, are possible under comparably oligotrophic conditions. Moreover, the high September N_2_ fixation rates coincided with the lowest DIN concentrations of all sampling occasions (0.20–0.29 μmol L^-1^). N_2_ fixation is energy-costly and many diazotrophs can increase their N_2_ fixation in times of inorganic/organic N scarcity (Mulholland et al., 2001). However, this potential negative relationship could not be evidenced with linear regression. On the contrary, a positive relationship was found during the mixed period while no relationship was found during the stratified period. A large part of the DIN measured during this study consisted of NO_x_. NO_3_^-^ in particular has a lower inhibitory effect on nitrogenase (the enzyme responsible for N_2_ fixation), compared to NH_4_^+^, depending on, e.g., light or PO_4_^3-^ availability (Dekaezemacker and Bonnet, 2011; Knapp, 2012; Garcia and Hutchins, 2014). Thus, certain abiotic factors may mitigate the nitrogenase inhibiting effects of NO_3_^-^. Alternatively, ambient DIN concentrations may not be high enough to inhibit nitrogenase (Mulholland et al., 2001). During the stratified period, when temperature was highest and the highest PAR values were measured, N_2_ fixation rates coincided with these parameters. However, when combined, PAR was the environmental parameter that best explained N_2_ fixation rates found throughout the year suggesting a prominent role for photoautotrophic diazotrophs. This coincides with relationships found for other organisms and substrates (Bednarz et al., 2015b; Tilstra et al., 2017).

**Table 5:** Comparison of hourly dinitrogen (N_2_) fixation rates from this study and literature references (given as ranges) measured by acetylene reduction assay.

Location	N_2_ fixation (nmol C_2_H_4_ L^-1^ h^-1^)	Reference
Gulf of Aqaba	0.07–1.28	This study
North Pacific Ocean	∼0.06–0.08	Mague, 1974
Central North Pacific Ocean	0.004–0.098	Mague et al., 1977
Central Arabian Sea	∼0.15	Capone et al., 1998
Western North Pacific Ocean	∼0.01–0.46	Kitajima et al., 2009
Northeast Atlantic Ocean	<0.03	Benavides et al., 2011
Hawaii	∼1.10–2.75^∗^	Wilson et al., 2012
Northern South China Sea	0.10–0.87	Wu et al., 2018

*^∗^assuming a 12:12 h light:dark cycle.*

**Table 6:** Comparison of N_2_ fixation and net primary production associated with different coral reef organisms and substrates from the Gulf of Aqaba.

Reef organism/substrate	N_2_ fixation activity			Net primary production			
	Mixed	Stratified	*p*	Mixed	Stratified	*p*	Reference
Unknown planktonic diazotrophs	8.07 ± 1.64	11.92 ± 4.34	0.423	-3.81 ± 0.98	-6.27 ± 0.36	**0.004**^∗^	This study
*Acropora* sp.	0.36 ± 0.11	4.65 ± 1.53	**0.002**^∗^	14.44 ± 0.78	13.14 ± 1.01	0.323	Cardini et al., 2015
*Stylophora* sp.	1.84 ± 0.45	5.71 ± 1.63	**0.027**^∗^	14.09 ± 1.13	10.77 ± 0.94	**0.0318**	
*Pocillopora* sp.	2.23 ± 0.44	3.64 ± 0.82	0.250^∗^	11.11 ± 1.32	11.93 ± 1.27	0.655	
*Goniastrea* sp.	1.78 ± 0.42	10.15 ± 1.93	**<0.001**^∗^	14.91 ± 1.11	14.58 ± 1.04	0.825	
Turf algae	53.10 ± 6.12	159.82 ± 29.09	**<0.001**^∗^	10.86 ± 0.52	14.27 ± 1.36	0.080^∗^	Rix et al., 2015
*Mycale* sp.	1.15 ± 0.34	7.77 ± 1.81	**<0.001**^∗^	-3.76 ± 0.50	-7.78 ± 1.05	**< 0.001**^∗^	
Xeniidae	0.17 ± 0.03	0.75 ± 0.20	0.184^∗^	10.16 ± 0.84	8.58 ± 0.49	0.114	Bednarz et al., 2015a
*Sarcophyton* sp.	0.89 ± 0.23	1.87 ± 0.48	0.154^∗^	4.88 ± 0.63	5.31 ± 0.79	0.665^∗^	
Carbonate sand	73.45 ± 15.96	65.52 ± 12.64	0.677^∗^	6.49 ± 1.63	4.23 ± 0.50	0.376^∗^	Bednarz et al., 2015b
Silicate sand	37.40 ± 6.01	35.50 ± 4.10	0.795	-	-	-	
Microbial mat	334.66 ± 51.50	241.92 ± 15.65	0.395^∗^	16.94 ± 1.51	15.51 ± 1.30	0.471	
*Caulerpa* sp.	24.36 ± 10.50	27.26 ± 4.48	**0.031**^∗^	23.75 ± 2.38	19.29 ± 1.29	0.116	Tilstra et al., 2017
*Lobophora* sp.	9.10 ± 3.20	33.81 ± 7.16	**<0.001**^∗^	7.73 ± 0.54	6.69 ± 0.45	0.144	

*Planktonic diazotroph N_2_ fixation and Net primary production in nmol C_2_H_4_ L^-1^ day^-1^ and μmol O_2_ L^-1^ day^-1^, respectively. All other values for N_2_ fixation and Net primary production are given in nmol C_2_H_4_ cm^-2^ day^-1^ and μmol O_2_ cm^-2^ day^-1^, respectively. Comparison data was obtained by averaging raw values of parallel investigated organism and substrates from winter and spring (mixed period) and those from summer and autumn (stratified period). Differences were tested with independent sample t-tests, except for p-values with ^∗^, these were tested with Mann–Whitney U-tests due to lack of normality. Bold p-values indicate p < 0.05.*

While GPP remained stable between both periods, the decrease in NCP found in the present study indicates that the planktonic community became more heterotrophic in the stratified period compared to the mixed period. However, this observation is contrasted by linear relationships (**Table 3**), which show that N_2_ fixation rates during the stratified period coincided with light availability (indicating autotrophic dominance) rather than DOC availability (not shown in **Table 3**) despite the higher availability of DOC. The benthos of the study site was primarily dominated by primary producers (see Cardini et al., 2016; van Hoytema et al., 2016), many of which release a significant fraction of their photosynthetically fixed carbon as DOC in the surrounding waters (Haas et al., 2010, 2011; Naumann et al., 2010). Since our water samples were taken 1 m above the benthos, DOC would have been available to be consumed by the bacterioplankton, as previously shown in Haas et al. (2011), potentially causing an increase in heterotrophic abundance and/or activity. While Chl *a* concentration per planktonic cell could have increased due to increased temperature (Geider, 1987) from the mixed to the stratified period, Chl *a* concentrations (measured per liter) found here decreased with increasing temperature, lending support that less autotrophs were present in the planktonic community during the stratified period. Moreover, Rahav et al. (2015) showed a shift toward a more heterotrophic diazotroph community from the mixed to the stratified period while Al-Najjar et al. (2007) also showed an increase in *Prochlorococcus* sp. abundance in the picophytoplankton community. Thus, it is likely that the planktonic community during the stratified period contained heterotrophic diazotrophs with lower N_2_ fixation rate capacity that was compensated for by higher abundances of *Prochlorococcus* sp. However, a full assessment of the planktonic community, including the identification of the picophytoplankton community, could shed light into this apparent contradiction. Additionally, the potential increase in *Prochlorococcus* sp. (Al-Najjar et al., 2007) may explain why a relationship between *P*_g_ and PO_4_^3-^ could not be established, despite DIN:PO_4_^3-^ ratios in the stratified period regularly exceeding Redfield (with values up to 33). Foster et al. (2009) also did not detect P limitation of N_2_ fixation and attributed this to the relatively small size of the N_2_ fixing microbes in the Gulf of Aqaba, allowing maintenance of N_2_ fixation at very low P availability. Small cell size theoretically results in increased nutrient uptake affinity due to allometrically higher surface area to volume ratio, which may have allowed *Prochlorococcus* sp. to maintain photosynthesis under extremely low PO_4_^3-^ availability (Finkel et al., 2010). However, nutrient limitation of planktonic processes is likely more complicated than indicated by the canonical Redfield ratio (Klausmeier et al., 2004).

Assuming the Redfield C:N ratio (6.625), planktonic N_2_ fixation had, on average, the potential to contribute 3.42% of the N needed for daily GPP during the mixed period. Remarkably, the average potential contribution during stratification was substantially higher as N_2_ fixation generated 20.84% of the potential N demand by GPP. This more than sixfold higher potential contribution indicates that N_2_ fixation is a substantial source of N for maintaining stable GPP by the autotrophic community as a whole during extremely oligotrophic conditions in the Gulf of Aqaba. Moreover, the percentage contribution calculated in the present study are similar to those under comparable oligotrophic scenarios in other regions (Montoya et al., 2004; White et al., 2007; Wu et al., 2018).

### Comparison With Parallel Investigated Organisms

Dinitrogen fixation and net photosynthesis of organisms and substrates were previously examined in a seasonal resolution in parallel with the current study (Bednarz et al., 2015a,b; Cardini et al., 2015; Rix et al., 2015; Tilstra et al., 2017). For comparisons, raw data of each organism or substrate was averaged after combining winter and spring data to represent the mixed period, and summer and autumn to represent the stratified period.

Comparing the pattern of planktonic N_2_ fixation rates between mixed and stratified periods with those of benthic N_2_ fixers revealed that, with the exception of *Pocillopora* sp. and both investigated soft corals (i.e., Xeniidae and *Sarcophyton* sp.), symbiotic diazotrophs (those with a eukaryotic host) are significantly affected by seasonal mixing and stratification, while planktonic diazotrophs were not (**Table 6**). The scleractinian coral *Pocillopora* sp. has recently been shown to have an inflexible microbiome in response to environmental stress (Pogoreutz et al., 2018). This could explain the deviation from the other three scleractinian coral genera as the diazotrophic community could remain relatively stable throughout the year. Furthermore, higher activity of resident diazotrophs is unlikely since abundance and activity of diazotrophs in Red Sea originating *Pocillopora* sp. have been shown to correlate positively (Pogoreutz et al., 2017). Strikingly, soft corals possessing symbiotic diazotrophs (Bednarz et al., 2015a), also show no differences between both periods (**Table 6**). Soft corals lack a hard calcium carbonate skeleton and a significant part of a soft corals’ weight can consist of water (∼80%; Al-Sofyani and Niaz, 2007). In this study, planktonic samples were taken 1 m above the reef benthos. Thus, soft corals can potentially take up water holding the same community of planktonic diazotrophs found here by replenishing their water content regularly (Sheppard et al., 2009). Together with its core coral microbiome (Ainsworth et al., 2015), which might be distinct from its surrounding seawater (Bourne et al., 2013), this could potentially alter their total diazotrophic activity to mimic that of the planktonic community described here. While a similar case could be made for the sponge *Mycale* sp., research has shown that between 72 and 93% of plankton is grazed and metabolized resulting in 74% of total daily C intake (Pile et al., 1996), while in corals, up to 95% of daily C is translocated from their photosynthetic endosymbiont *Symbiodinium* (Muscatine, 1990) suggesting a low need for grazing the diazotrophs that are taken up with the surrounding water.

Particulate nitrogen of all investigated organisms and substrates remained relatively stable with the exception of *Stylophora* sp. and *Mycale* sp. The latter due to high respiration rates measured during summer and autumn (Rix et al., 2015). In general, the pattern of *P*_n_ of the planktonic (free living) diazotrophs differed from the benthic (symbiotic) diazotrophs (**Table 6**).

### Concluding Remarks

Primary production in the water column directly overlying a coral reef in the Gulf of Aqaba appears to be primarily regulated by inorganic N availability, driven by mixing conditions. While inorganic N concentrations declined due to stratification, daily contribution of planktonic GPP to TOC declined significantly while GPP itself remained relatively stable. Compared to open water GPP (Rahav et al., 2015), GPP measured here was between three- and ninefold higher suggesting potential benthic coupling. NCP was significantly more heterotrophic during stratification. However, the daily contribution of NCP to TOC was similar between the two periods due to increased DOC concentrations. The maintenance of biological activity in the water column due to increased DOC availability in times of reduced GPP highlights the importance of the microbial loop in planktonic trophodynamics in these waters (Azam and Malfatti, 2007; Nelson et al., 2011).

In addition to the change in the C budget, there were indications of a potential shift in the N_2_ fixation community toward higher heterotrophic activity, similar to findings of Rahav et al. (2015). Causes for this shift could be the decline in inorganic nutrients as well as the increased DOC concentration providing a competitive advantage to heterotrophic diazotrophs (Suggett et al., 2009). This increase in DOC could be attributable to increased release by benthic turf- and macroalgae (Haas et al., 2013; Mueller et al., 2014). Algal-derived organic matter may also promote a more heterotrophic planktonic community than organic matter released by hard corals (Haas et al., 2011; Nelson et al., 2013). During stratification, when GPP is strongly nutrient-limited, N_2_ fixation shows the potential to contribute a substantial fraction of the N needed, by compensating for low DIN, to maintain stable GPP in the water column.

Dinitrogen fixation was maintained at comparable rates in both periods. Moreover, the results obtained here strongly suggest that N_2_ fixation is an important source of N to planktonic primary production. In addition, DOC appears to play an important role in the dynamics of planktonic C and N production/consumption. Further investigation into DOC dynamics through coral reefs is warranted to unravel its effect on energy and nutrient cycles in coral reefs and their surrounding waters. Finally, the findings presented here may be applied to lower latitude coral reefs where the more stable environmental conditions make the disentanglement of driving environmental parameters more complicated.

## Author Contributions

AT analyzed data, wrote the manuscript, prepared the figures and tables, made revisions, and in charge of submission. NH wrote part of the manuscript, reviewed drafts of the manuscript, and designed and performed the experiments. UC, VB, LR, and MN reviewed drafts of the manuscript and designed and performed the experiments. FA-H contributed reagents, materials, and analysis tools, and designed the experiments. CW reviewed drafts of the manuscript and designed the experiments.

## Conflict of Interest Statement

The authors declare that the research was conducted in the absence of any commercial or financial relationships that could be construed as a potential conflict of interest.
